# A Forty-Eight Hour Week

**Published:** 1919-12-13

**Authors:** 


### THE NATION AND THE NURSE.

### A Forty-Eight Hour Week.

With your permission, Mr. Editor, I want to have a word with Lord Knutsford, but before so doing may I shake hands with your ingenuous correspondent "F. A. S.," of 14 St. Thomas's Street? "Leave the woman in the wards," she naïvely observes, "to her matron and Committee."

"F. A. S." hails from St. Thomas's Street. A good many years ago, when I knew nothing of nurses, or nursing, or sick people, St. Thomas's Hospital was shown to me by my father as one of the sights of London. I saw many of the other wonders of the world then also, and, looking backward, I can honestly say that none impressed me so much as this magnificent pile of buildings, erected in the best part of London for the benefit of the sick poor. I felt, indeed, that it was a privilege to be sick in this place, and to hear Big Ben chiming the hours. St. Thomas's Hospital, its building, its equipment, its maintenance, must have cost millions. It is one of the finest of England's monuments, and somebody says that the character of a nation may be judged from its monuments.

Lord Knutsford's "Impossible."

I have always been an admirer of Lord Knutsford, though everybody may not think so. He is head of one of the greatest hospitals in the Kingdom. It is not on this account that I admire him, but because he says exactly what he thinks. He has the courage of his convictions, an attribute which is not common. You always know where you are with Lord Knutsford.

Now, in last week's Hospital, it is published that Lord Knutsford said that "a forty-eight-hours' week for nurses is impossible." And he gave his reasons—"Patients will need to engage three nurses instead of two." And "think what it would mean for a woman in her confinement to have three nurses instead of one."

This argument is based on the assumption that general nurses work for twelve hours per day at present, and maternity nurses for twenty-four. I do not believe that any man or woman is physically able to work these hours, and I do not believe that nursing which is calculated to exhaust the nurse is good for the patient. No hospital is justified in taking in more patients than a reasonably worked nursing staff can look after.

The next argument is, "We cannot afford it. . . . Where could the nurses be housed, and how could they be paid," My answer is this. Just walk down the Thames Embankment and look at St. Thomas's. England can afford that. Am I to be told in all seriousness that England cannot afford to pay and house her nurses? Lord Knutsford has the attribute of being a plain, blunt man. May I ask him a plain, blunt question. For whom are the Ministry of Health and the Ministry of Reconstruction contemplating the expenditure of vast sums of money for housing? Let us get down to hard facts. I should immensely like to know exactly what Lord Knutsford thinks of the Englishman's attitude in the matter of paying for the nursing of his own sick poor. Is it his desire that the shop girl, the factory wench, the typist, the schoolmistress, the cook, the dairymaid, and any others you may think of shall have a better time than the hospital nurse? If this is what my countrymen think I do not think much of them. But Lord Knutsford's experience is vastly greater than mine, and he would make his statement more understandable if he would state exactly on what grounds he bases the opinion that the nation will refuse to provide reasonable pay and housing accommodation for hospital nurses. This, I take it, is the real problem.

If "F. A. S." has not gone away, I should like to add a parting word. Her letter to the Editor is on page 219. On page 221 is an interesting article describing how £100,000 is to be distributed. It is a Red Cross grant—surplus funds. I have read this article very carefully, and I have re-read it. Not a word about the nurse! Have you noticed it, "F. A. S."? During the war the nurse was everybody. The nurse was bombed. The nurse went down in the hospital ship. The nurse was filmed, haloed, flattered. Now, if you please, she cannot be housed. To my mind this £100,000, every shilling of it, ought to have gone to the nurses. They were in reality the Red Cross, and the surplus funds ought to have been devoted to their benefit. Did the College of Nursing enter a claim on their behalf? I wonder.

[Ierne lacks the gift of patience. £500 was given By the Red Cross Society to the Dundee Centre of the College. There is more to come. See page vi.—Ed. The Hospital.]

Are all you ladies and gentlemen who do not like what I say blind? Are your heads buried in the sand? Or have I taken leave of my senses? IERNE.

